# Additive Manufacturing of Metals Using the MEX Method: Process Characteristics and Performance Properties—A Review

**DOI:** 10.3390/ma18122744

**Published:** 2025-06-11

**Authors:** Katarzyna Jasik, Lucjan Śnieżek, Janusz Kluczyński

**Affiliations:** Institute of Robots & Machine Design, Faculty of Mechanical Engineering, Military University of Technology, Gen. S. Kaliskiego 2 St., 00-908 Warsaw, Poland; lucjan.sniezek@wat.edu.pl (L.Ś.); janusz.kluczynski@wat.edu.pl (J.K.)

**Keywords:** 3D printing, additive manufacturing, material extrusion, metal composites, metals, performance properties

## Abstract

Compared to traditional manufacturing methods, additive manufacturing (AM) enables the production of parts with arbitrary structures, effectively addressing the challenges faced when fabricating complex geometries using conventional techniques. The dynamic development of this technology has led to the emergence of increasingly advanced materials. One of the best examples is metal–polymer composites, which allow the manufacturing of fully dense components consisting of stainless steel and titanium alloys, employing the widely available AM technology based on material extrusion (MEX). Metallic materials intended for this type of 3D printing may serve as an alternative to currently prevalent techniques including techniques like selective laser melting (SLM), owing to significantly lower equipment and material costs. Particularly applicable in low-volume production, where total costs and manufacturing time are critical factors, MEX technology of polymer–metallic composites offer relatively fast and economical AM of metal components, proving beneficial during the design of geometrically complex, and low-cost equipment. Due to the significant advancements in AM technology, this review focuses on the latest developments in the additive manufacturing of metallic components using the MEX approach. The discussion encompasses the printing process characteristics, materials tailored to this technology, and post-processing steps (debinding and sintering) necessary for obtaining fully metallic MEX components. Additionally, the article characterizes the printing process parameters and their influence on the functional characteristics of the resulting components. Finally, it presents the drawbacks of the process, identifies gaps in existing research, and outlines challenges in refining the technology.

## 1. Introduction

Additive manufacturing (AM), commonly referred to as 3D printing, is a production technique in which physical three-dimensional objects are constructed through the successive deposition of material layers, generally guided by computer-aided design (CAD) data. Presently, there are over 50 different types of 3D printing technologies, categorized according to ISO international standard 17296-2 [1,2] into Powder Bed Fusion (PBF), Material Jetting (MJ), Binder Jetting (BJ), Directed Energy Deposition (DED), Sheet Lamination (SL), Vat Polymerization (VP), and Material Extrusion (MEX). Due to the coexistence of various terms in the literature referring to extrusion-based AM processes, it is herein established that the terms Fused Filament Fabrication (FFF) and Bound Metal Deposition (BMD) are used interchangeably with Material Extrusion (MEX). Among these methods, MEX is widely employed across various industries due to its advantages, including the widespread availability of devices and dedicated materials, low cost compared to other AM methods, and relatively straightforward device operation [3,4,5,6,7,8]. The cost-effectiveness of MEX technology makes it particularly attractive for producing small-batch or customized metal parts, as the equipment and material expenses are significantly lower than those of laser-based AM techniques [9,10]. Improvements in binder formulations and process optimization have enhanced the densification of MEX-manufactured metal parts, enabling better control of porosity and mechanical properties, which were previously key limitations [11,12]. Additionally, MEX processes have been shown to reduce material waste and energy consumption compared to traditional subtractive manufacturing and some other AM techniques, contributing to more sustainable production methods [13].

AM enables the production of highly functional structures characterized by both lightweight and stability, achievable through structural optimization facilitated by AM technology. Consequently, designers can produce components with optimized topology, minimizing material consumption while ensuring the required strength. Parts manufactured using AM are lighter than their counterparts produced using traditional methods without compromising mechanical properties, meeting specific functional requirements. Moreover, the costs associated with manufacturing parts using AM are largely independent of the quantity produced, as there is no need to invest in expensive tools and molds [14]. Therefore, AM is well-suited for producing small quantities of unique components. This technology finds applications in various fields beyond prototyping, including modeling, tool production, and the manufacturing of end-use parts [15]. The rapid growth in the development of AM has led to its widespread adoption in various industrial sectors such as medicine, aviation, automotive, and energy [16,17,18,19]. Its versatility has made it an integral component of novel approaches across diverse domains, offering novel opportunities for design and manufacturing [20].

Following polymers [21,22,23,24,25], metals are the primary materials employed in AM, with research in this domain spanning nearly two decades [26]. Metals in AM exhibit notable characteristics distinguished by their high-fidelity microstructural alterations amidst elevated temperatures, ensuring that the resulting metallic components possess requisite mechanical attributes and dimensional precision to fulfill the specifications of their intended application. Research on metallic materials [27,28,29,30,31,32] has found applications in various AM technologies [33], including selective laser sintering (SLS) [34,35,36,37], direct metal deposition (DMD) [38,39,40], shaped metal deposition (SMD) [41,42] and selective laser melting (SLM) [43,44,45,46]. While the previously mentioned metal AM technologies are popular, their production costs are often high, influencing the final product prices [47,48]. An alternative method is metal printing using the MEX technique, which enables the fabrication of various metals such as stainless steel, titanium alloys, cobalt alloys, aluminum alloys, and nickel-containing alloys [49,50,51,52,53,54]. Metal printing using MEX is progressively investigated due to its advantages, including cost-effectiveness and user-friendly equipment [55,56,57]. Nevertheless, the complexity of the MEX process presents challenges related to forming, binder removal, and sintering, influencing the quality and functional properties of the final printouts [58,59]. In the context of material extrusion of polymeric-metal composites (MEXM) processing, typical drawbacks include dimensional inaccuracies, potential delamination, surface roughness, porosity, deformations, and anisotropic mechanical properties. Thorough analysis and research are necessary to minimize flaws and expand the potential applications of this technology in more demanding manufacturing areas.

The main objective of this article is to provide a comprehensive review of ongoing research on the functional properties of metal–polymer composites 3D printed using the MEXM technique. This study provides a comprehensive characterization of the techniques and materials used in metal-based MEXM, describes the post-processing stages necessary to obtain fully metallic parts, and examines the influence of individual process parameters, part orientation, and infill type on the final properties of the components. A comparison is also made between MEXM, which is a more widely employed method for producing metal parts, and other techniques such as SLM and SLS. Finally, the article concludes with a summary of existing research and outlines perspectives on the further development of MEXM technology.

## 2. Characterization of the Printing Process and Applied Materials

### 2.1. Materials

In the case of MEXM technology, the metallic material takes the form of a composite consisting of well-dense metallic powder and a polymer binder. To obtain fully metallic printed parts, known as green parts (GP), the components must undergo a catalytic debinding process, which involves partially removing the main part of the polymer (polymeric binder) [60]. As a result, a so-called brown part (BP) is obtained, comprising particles of pure metal and residual binder (secondary binder) [61,62]. The final step involves sintering at temperatures slightly below the metal’s melting point, which removes the secondary binder from the brown part (BP), causes the metal particles to fuse, and results in a fully metallic part [63,64]. The composite filament exhibits a wide range of compositional formulations depending on the powder content, typically ranging from 50% to 65% by volume, though in some cases it can reach up to 80% [65]. The percentage of powder directly affects the sintering process, as a higher percentage leads to a greater density of the final element. However, too little powder can result in deformations during the processing of BP. This content also influences the mechanical and tribological properties of the material [66]. The most-used materials for composite production include stainless steel, titanium alloys, aluminum alloys, nickel-based alloys, as well as chromium and cobalt alloys [67]. The percentage composition of these materials varies depending on the manufacturer. Table 1 presents the most frequent materials with the percentage contribution of individual elements in the powders’ chemical composition.

The binding system most commonly appears in the form of a polymer and consists of a base material, a framework, and additives, which are proportionally distributed differently depending on the material. The base material constitutes 50 to 90% of the volume of the binding system, the framework does not exceed 50%, while the remaining additives make up a maximum of 10%. Various types of binding systems are employed in composites, influencing material rheology, solvent removal, as well as microstructure and porosity. When the binding system constitutes a high percentage of the volume, the flow intensity of the alloy will also be high. Table 2 presents examples of binding materials with their compositions.

The selection of a polymeric binder system for metal-extrusion-based manufacturing processes such as MEXM is critically dependent on the specific properties and processing requirements of the target metal or alloy. Binder systems must fulfill several essential roles: they provide mechanical strength to the filament, allow consistent extrusion, facilitate shape retention during printing, and must be fully removable without residue prior to sintering. Different metals may require different thermal and rheological properties from the binder system. For example, binders for titanium alloys often require stricter oxygen control during processing, necessitating clean-burning polymers or specialized components with lower residual content after debinding. Stainless steels, such as 17-4PH or 316L, are more tolerant to certain additives but still require binders with predictable thermal degradation behavior and sufficient flowability. The reason for limiting additional components—such as plasticizers, surfactants, or flow agents—to no more than 10% of the total binder content lies in maintaining the functional integrity of the main binder. Excessive additives can compromise mechanical properties of the green part, induce phase separation, or lead to undesirable residue after debinding, which would negatively affect sintering and final mechanical properties. Moreover, higher concentrations of additives often reduce viscosity stability and may interfere with printing consistency or dimensional accuracy of the parts. Therefore, limiting additives to a maximum of 10% helps ensure a balance between flexibility and structural integrity of the filament, while reducing the risk of defects such as porosity, delamination, or uneven shrinkage in the final sintered part.

### 2.2. Manufacturing Process—GP

**Figure 1:**
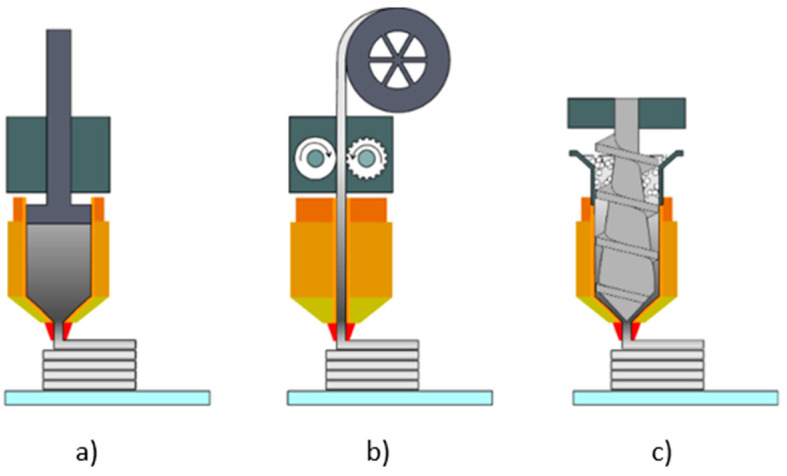
Types of printers used in MEXM technique: (**a**) plunger-based type, (**b**) filament-based type, (**c**) screw-based type [74]).

Metal part production through the MEXM technique is carried out using standard devices dedicated to this method. Depending on the material delivery system, three types of printers working with the MEXM technique are distinguished, as illustrated in Figure 1—(a) plunger-based type, (b) filament-based type, and (c) screw-based type [73].

The most prevalent and extensively utilized metallurgical procedures in MEXM involve filament-based methods, frequently known as Fused Deposition Modeling (FDM) or Fused Filament Fabrication (FFF) [75]. Polymer-ceramic filaments cannot be too brittle due to the occurrence of resistance during the transition from the cooled to the hotend zone of the extruder. To reduce brittleness, these filaments are coated with other materials to mitigate this property. Within the MEXM process, the metal filament is conveyed through the extruder system to the hotend and heated nozzle, where it undergoes melting and is sequentially deposited layer by layer by the generated operational file (g-code). This approach offers advantages such as safety, simplicity, low equipment expenses, and compatibility with standard 3D printers. However, the substantial metal proportion in the filament necessitates a specialized nozzle made of hardened steel or ruby to ensure consistent material flow and prolong the nozzle’s longevity [76]. The filament not only traverses the extruder rollers in opposing motion but also acts as a piston, exerting pressure towards the nozzle. Consequently, the filament must possess adequate strength, rigidity, and low viscosity to mitigate the risk of cracking or deformation upon passage through the extruder mechanism [77]. Furthermore, the material should demonstrate robust adhesion to effectively bond with the preceding layer or substrate surface. Additionally, a high modulus of elasticity and bending strength are essential for the orderly spooling of the material and maintaining a continuous filament flow to the printhead during printing [78]. Moreover, to fulfill these criteria, the material must exhibit high load-bearing capacity to accommodate maximum metallic powder content [79]. Precision in printed and sintered parts largely hinges on meticulous adjustment of MEXM process parameters, tailored, and optimized for specific scenarios, accounting for geometric model intricacies, material characteristics, and the shape and dimensions of the printed components [80,81]. Several adjustable parameters are critical, including print temperature, perimeter count, infill pattern, print speed, flow rate multiplier, and layer thickness [82]. Print temperature is influenced by the type of binder, metal powder, powder particle loading, and printing speed, typically maintained within the range of 80 to 260 °C. This temperature variation is contingent upon the binder type, with water-soluble binders requiring lower temperatures compared to those necessitating solvent and/or thermal treatment. The heated print bed, operating within the range of 40–100 °C, plays a pivotal role in enhancing adhesion and minimizing deformation induced by shrinkage during solidification [83], a widely adopted practice in MEXM technology with polymers.

Considering the subsequent debinding and sintering stages, CAD models must be dimensioned with the anticipated isotropic shrinkage that occurs during densification. For 17-4PH and 316L feedstocks, linear shrinkage of 14–23% has been reported; an overall scale-up factor of 1.16–1.24 is therefore recommended [84,85]. Green walls thinner than 1.2–1.5 mm (corresponding to two perimeters when printing with a 0.4 mm nozzle) tend to warp or densify incompletely, so this threshold should be observed to guarantee mechanical integrity [86]. Overhangs steeper than 45° can generally be built without additional structures, whereas shallower angles, large flat undersides, and internal cavities require removable metal scaffolds or a ceramic release layer. Lattice or tree-style supports are preferred over solid pillars because they lower thermal mass and permit more uniform free shrinkage, thus reducing distortion. Wherever possible, support should be attached to non-critical faces or machining allowances to avoid surface blemishes on functional features [87]. Table 3 presents the print parameters for selected materials.

Furthermore, diverse adhesive coatings are applied to the print bed. Conversely, closed commercial systems employ a raft, a printed layer positioned between the component and the bed, to improve the bonding strength of the first layer. Printing parameters, including speed and flow rate multiplier, wield a pivotal influence on the quality of raw components [90]. Achieving high print density is recommended by executing outlines. Additionally, layer thickness and cooling methods influence the performance properties of the produced components. Ren et al. [91] noted that the tensile strength of printed MEXM is primarily affected by the following parameters in descending order: infill percentage, raster angle, and layer thickness. Also, Obadimu et al. [92] demonstrated that the raster angle significantly influences the mechanical properties of components, along with layer height, while print speed exerts no discernible influence on the final properties. During the post-processing phase, dimensional alterations manifest, prompting the need to contemplate the potential shrinkage of previously manufactured metallic components during the design phase [93]. The authors [94] highlight that the linear shrinkage of samples is contingent upon the printing orientation. Samples printed parallel to the build platform plane exhibited approximately 20% shrinkage, while those printed perpendicularly experienced a shrinkage of 25.20%. The precise values obtained are detailed in Table 4.

**Figure 2:**
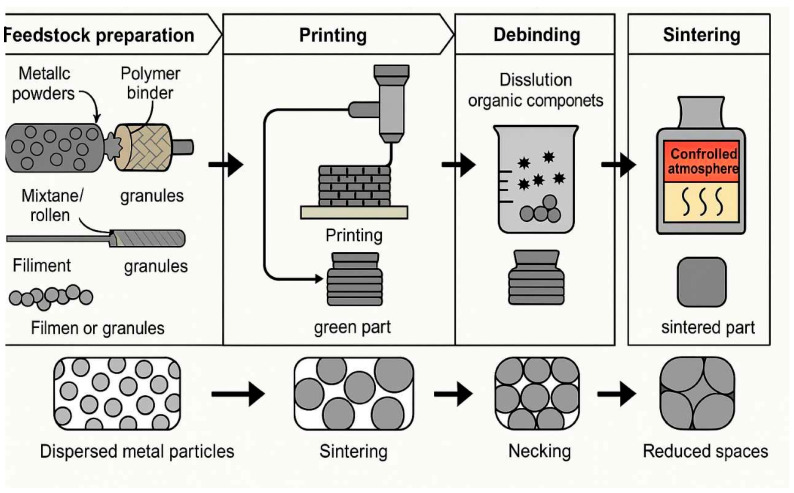
Key stages and parameters in the synthesis of metallic materials via additive manufacturing (AM) technology.

Figure 2 illustrates the key stages and parameters involved in the synthesis process of metallic materials using AM technology.

### 2.3. Debinding—BP

GP must undergo the debinding process, which involves the elimination of the binding material. The polymeric binder is potentially removed in various ways, featuring the greatest common methods employing solvent removal, catalytic removal, and thermal removal. These processes differ in duration and controllability. The performance of debinding is also influenced by the thermal cycle, the type of bond removal, and the composition of the binding agent used, which affects print quality. Temperature control during the removal process is crucial to avoid undesirable effects. Thermal processing is the most used. The procedure entails heating the printed components to temperatures between 60 °C and 600 °C, depending on the chosen solvent, which may include heptane or trichloroethylene [84]. Subsequently, the prints are heated at the target temperature for a specified time, which depends on the raw material. However, the applied temperature must not be too high to prevent material binder decomposition into carbon. Table 5 presents debinding parameters for available materials.

The debinding process involves intricate stages of solvent extraction and thermal removal, meticulously considering the precise diffusion and dissolution processes of various components within the debinding system [85]. An integral aspect of this process entails the thorough elimination of all binder components, ensuring the production of high-quality prints that accurately replicate their entire geometric shape. Strategic selection of the removal process and careful control of thermal cycles are paramount to preserving print integrity and minimizing thermal stresses. Lotfizarei et al. [86] conducted microscopic analyses to investigate how the debinding speed influences the porosity of components produced from 316L steel. Printed samples underwent heating to 250 °C at a rate of 5 °C/min, proceeded by further heating to 400 °C at rates of 0.2, 0.7, and 1 °C/min. Monitoring was maintained until mass variations ceased entirely. Thereafter, the components were subjected to sintering at 1360 °C for a duration of 6 h to facilitate porosity evaluation. Notably, samples subjected to lower debinding degrees exhibited higher relative density, showcasing a marked improvement in porosity with decreasing debinding rate. Wagner et al. [87] analyzed the role of different stages of the debinding cycle on binder removal. The results demonstrated that a dual-stage approach, comprising both solvent and thermal debinding, efficiently removed the binder. The binder, stearic acid, underwent full thermal decomposition between 160 °C and 260 °C, while thermal breakdown ensued in the temperature interval between 300 and 460 °C. Examination of the influence of heating rate on microstructure highlighted the significant role of binder content in defect formation within the sample. Moreover, studies by various authors [95,96] delved into the influence of binder removal at different temperatures. Results revealed that higher temperatures facilitated a swifter breakdown of polymer binder particles. Furthermore, meticulous matching of the solvent with the primary binder during raw material development emerged as crucial for successful binder removal. Given the time-intensive nature of this process, it is imperative for the main binder to readily dissolve in the applied solvent, thereby reducing process duration. While elevated debinding temperatures may expedite the process, caution must be exercised to avoid adverse effects on the primary binder responsible for binding the metal powder [97,98].

### 2.4. Sintering—Fully Metal Parts

**Figure 3:**
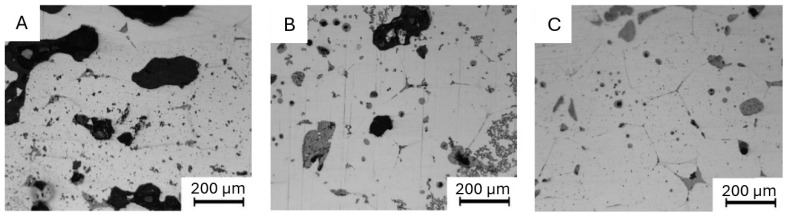
Effect of sintering temperature on the relative density of samples for (**A**) 1 °C/min; (**B**) 0.7 °C/min and (**C**) 0.2 °C/min [9].

The final stage of processing involves sintering, a process that facilitates the comprehensive removal of the bonding material from the BP by consolidating the metallic powder to achieve a densely packed structure. During this processing stage, significant shrinkage occurs, leading to an increase in theoretical density of up to 99%. Sintering entails the rearrangement and mass transport of metallic particles, involving vaporization and condensation, surface, and volume diffusion followed by plastic flow between adjacent powder particles. Shrinkage during the sintering process can range from 12% to 20%, which is influenced by various factors, primarily the type and size of metallic powder particles, the content and distribution of powder, sintering temperature, and duration [99]. As the process progresses, particles gradually strengthen their bonds, thereby eliminating pores. The final form of the obtained parts is a solid, dense structure with a density ranging from 95% to 99.5%. Simultaneously, significant material shrinkage occurs during the sintering process. The process temperature is maintained slightly below the material’s melting point to prevent liquefaction. Despite the occurring shrinkage, maintaining optimal temperature and appropriate processing time allows for the preservation of the part’s shape and geometric features [83]. Gloeckl et al. [100] showed that the relative density of sintered Ti-6Al-4V alloy obtained via MEXM technology increases with rising temperature and sintering duration. The authors further noted that employing fine powder enhances diffusion, induces greater shrinkage, and results in a higher relative sintered density in comparison to coarser powder particles. However, excessive increases in temperature and sintering time can result in significant grain growth, adversely affecting mechanical properties. Therefore, during sintering, it is essential to adjust the process temperature and duration to obtain an optimal combination of these values, ensuring high sintered density without compromising mechanical properties. Authors [9] observed that maintaining an optimal sintering temperature over an extended duration, in combination with slower heating and cooling rates, produces components with higher density, as illustrated in Figure 3.

This effect is attributed to a more controlled solid-state diffusion mechanism, which promotes better particle coalescence and pore reduction. Slower heating rates minimize internal stress and reduce deformation caused by rapid thermal expansion. As a result, the final structure demonstrates higher homogeneity and relative density, which was confirmed through microstructural analysis and porosity measurements.

Each metallic alloy requires different sintering conditions, including temperature and sintering times, which are shown in Table 6.

The process atmosphere, alongside heating rate, temperature, and process duration is a crucial factor in heat treatment. It significantly influences the final properties, such as microstructure, porosity, size, and ductility of the AM-ed components [10]. The process can be conducted in the atmosphere of various gases, with argon, hydrogen, and nitrogen being the most common. In some cases, especially for stainless steel or certain titanium alloys, a vacuum atmosphere is preferred. Optimizing sintering parameters allows the achieving of fully dense metal part structures with a density approaching 99%. After the sintering process, parts can undergo various finishing treatments, such as machining or additional heat treatment. From a thermodynamic standpoint, densification during MEXM sintering is driven by the reduction in the total surface free energy of the powder compact. The initial neck-growth stage proceeds through surface diffusion, whereas intermediate and final stages are controlled by lattice and grain-boundary diffusion, both exhibiting Arrhenius-type temperature dependence (Q ≈ 290 kJ mol^−1^ for 316L). At 1300 °C this translates into a theoretical densification rate of c. 1.5% h^−1^, which matches dilatometry data obtained for the feedstock used in this study [101]. Kinetically, complete removal of the POM-based primary binder follows a Fickian diffusion law; keeping the debinding ramp below 0.7 °C min^−1^ avoids internal pressure build-up and micro-cracking [102]. The subsequent solid-state sintering step reaches > 96% relative density after 2–3 h at 1340 °C (316L) or 1310 °C (17-4PH). These values compare well with molecular-dynamics predictions for powder particles of 8–15 µm, confirming that the selected thermal cycle is both thermodynamically feasible and kinetically efficient [103].

**Figure 4:**
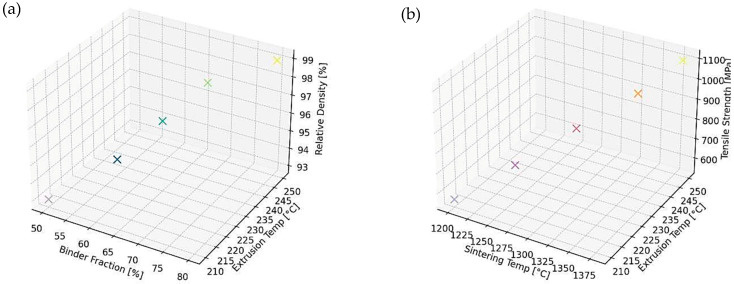
The graphs illustrate (**a**) the relationship between binder fraction and extrusion temperature and their typical impact on relative density, and (**b**) the effect of annealing and extrusion temperatures on tensile strength.

To provide a clearer overview of how the key processing variables affect the final material performance, a graphical summary has been developed and is shown in Figure 4.

### 2.5. Functional Properties of MEXM

MEXM in the production of metal parts is continuously advancing, with the introduction of new materials, although they have yet to undergo extensive analysis. Presently, the predominant materials utilized for printing and under scrutiny are primarily steel. Nonetheless, alternative materials including titanium alloy, nickel, copper, and aluminum alloys are also penetrating the commercial market. The selection of a specific alloy hinges on the project’s prerequisites, encompassing mechanical properties, chemical resistance, or thermal properties. Depending on the chosen alloy, various combinations of these properties can be achieved, facilitating customization for specific applications. Stainless steel offers commendable mechanical properties, corrosion resistance, and elevated durability. Titanium alloys furnish superior strength with reduced density and mass of components. Nickel alloys confer resistance to high temperatures, corrosion, and oxidation. Copper alloys exhibit the highest electrical conductivity alongside good thermal conductivity, whereas aluminum is suited for applications where mass reduction is paramount [104,105,106,107]. Given the intricacies of the metal part manufacturing process employing MEXM technology, each production stage assumes critical importance and can profoundly influence the resulting properties of the fabricated parts. The primary factors influencing the properties of metal parts manufactured via MEXM are delineated by the ASTM F3122-14 standard [108], which addresses the mechanical properties of metal parts produced using AM technology. Table 7 outlines the principal parameters influencing the characteristics of components manufactured via the MEXM method.

While many factors influence the final properties, MEXM steel manufacturers provide mechanical properties that can be achieved with their materials. These can be found in Table 8.

The industrial relevance of MEX-printed metals is already demonstrated in several sectors. 17-4PH parts such as quick-change gripper jaws or injection-mold inserts routinely withstand cyclic loads above 700 MPa after H900 aging, while retaining corrosion resistance comparable to wrought stock [111]. Austenitic 316L is favored for fluid-handling manifolds in food-processing lines, where its chloride resistance and printable internal channels shorten CIP times by 30% [101]. Patient-specific Ti-6Al-4V spinal cages produced by material extrusion meet ISO 5832-3 tensile requirements and show bone-ingrowth thanks to integrated lattice porosity [112,113]. Finally, fully dense copper heat-sinks printed via MEX achieve thermal conductivities > 380 W m^−1^ K^−1^, enabling > 15% higher heat-flux removal than conventional skived fins [114]. These case studies underline the practical maturity of the process and fill the identified gap concerning real-world applications.

## 3. Comparison of Metal Parts Manufactures by MEXM with Other Techniques

In the field of high-technology metal component manufacturing technologies, there is a burgeoning interest in the MEXM, prompting researchers to conduct rigorous comparative analyses with traditional methods like SLM. Kedziora et al. [115] conducted a study comparing the strength properties of stainless steel 316L and 17-4 PH produced using MEXM with 316L steel produced using the SLM technique. They examined parameters such as tensile strength, fatigue, and impact toughness. The authors noted a significant decrease in fatigue strength and tensile strength of MEXM-printed samples compared to counterparts obtained via the SLM technique. Defects arising during extrusion caused empty internal spaces within the structure, compounded by notable surface roughness influencing the deterioration of mechanical properties in MEXM printouts.

In another study, Gong et al. [116] compared 316L stainless steel printed using both MEXM and SLM techniques Microstructure analysis revealed fully dense SLM-printed samples with porosity below 0.1%, while MEX-printed samples exhibited a porosity of approximately 1.5%. Mechanically, MEXM parts demonstrated lower yield strength, tensile strength, and modulus of elasticity compared to SLM parts. Additionally, the elongation at break for MEXM was significantly lower than for SLM steel samples, with SLM samples also exhibiting higher hardness.

In a subsequent study published by the authors [117] the comparison involved 316L steel printed using MEXM and SLM and conventional techniques. Consistent with previous findings, steel printed using SLM and conventional techniques exhibited improved structural features, including reduced porosity, higher microstructural density, and increased hardness compared to MEXM-processed steel. In [118] the authors observed that despite the lower strength of MEXM steel components compared to those produced by SLM, MEXM samples exhibited sufficient mechanical properties for many practical applications. They found that MEXM components demonstrated comparable strength to SLM components, suggesting that MEXM use 316L steel, which could serve as a viable alternative for metal component manufacturing. The results imply that, despite certain limitations, MEXM could emerge as a competitive and cost-effective alternative to conventional metal component manufacturing methods [119]. In the other study [120], the authors discussed the production of MEXM filaments to produce Ti-6Al-4V titanium alloy parts. They successfully produced filaments with a 59% volume fraction of Ti-6Al-4V powder, featuring even powder particle dispersion and fluidity crucial for maintaining consistent extrusion during printing, which leads to defect-free microstructures and maximized print density. Employing a dual-stage approach for binder removal, encompassing solvent extraction and thermal degradation of the polymer binder, succeeded by vacuum sintering, yielded nearly isotropic shrinkage of approximately 14% in all dimensions, thereby achieving a sintered density of 94.2 ± 0.1%. The characteristics properties of the obtained Ti-6Al-4V alloy parts included a tensile strength of 875 ± 15 MPa, yield strength of 745 ± 10 MPa, and elongation at break of 17 ± 3%. Dimensional analysis, tolerance, and microstructure analysis confirmed the effectiveness of the MEXM process in producing complex and durable metal parts, suggesting promising potential for this technology, with the obtained results comparable to parts produced using MIM technology.

The above studies show that metal parts produced by the MEXM technique exhibit 370 MPa worse mechanical properties compared to counterparts produced using SLM technology 371 MPa or conventional manufacturing methods. One of the main reasons for the deteriorated properties is characterized by metallurgical defects, including porosity. Additionally, worsened mechanical properties may result from rapid temperature changes, capillary forces, as well as the influence of gravity in the absence of external pressure on bonding mechanisms, shrinkage occurring during the finishing process, and insufficient particle settling [121,122]. However, as shown by manufacturers (Table 9) of MEXM composite materials, higher values of utility properties can be achieved, but more research is needed to minimize the arising defects.

In comparison with other additive manufacturing techniques, MEXM exhibits both technological and economic advantages, yet also important limitations. Its key strengths include lower equipment costs, ease of operation, and material availability, which make it particularly suitable for rapid prototyping and small-batch production. Additionally, MEXM allows the use of composite and experimental filaments, enabling flexible material development. However, in contrast to techniques such as SLM or MIM, MEXM is characterized by a higher tendency to generate porosity, reduced density of sintered parts, and lower mechanical performance. These drawbacks stem from the nature of the extrusion-based deposition and the limitations of the debinding and sintering processes, which often cannot ensure complete removal of binder or full densification. As a result, the achievable dimensional tolerances, surface quality, and reproducibility are also inferior. Despite these limitations, for many non-critical applications where ultra-high mechanical performance is not required, MEXM remains a cost-effective alternative. The integration of additional processes such as heat treatment or pressure-assisted sintering can enhance the final properties, although this increases process complexity and time. Therefore, the use of MEXM should be carefully considered depending on the specific functional and mechanical requirements of the end-use application.

## 4. Effect of Process Parameters on Functional Characteristics

The optimization of printing settings is crucial to achieving the desired properties of produced parts. Products that attain the highest relative density, as well as high print quality without defects, exhibit better physical and mechanical properties. Despite the provision of parameter combinations by most manufacturers of metallic polymer composites dedicated to MEXM, ensuring the production of parts with commendable functional properties, practical outcomes often deviate from theoretical expectations. The selection of printing parameters frequently hinges not only depending on the fundamental attributes of the material but also on the models of printers employed for part fabrication. Numerous studies delve into the ramifications of printing parameters on the ultimate properties of parts, with bed and printing temperatures, layer thickness, and flow rate multiplier being commonly scrutinized variables [132]. Researchers vary these parameters in their studies, obtaining different combinations of properties depending on their specific focus. Table 10 presents a compilation of available studies where authors compare various printing parameters.

### 4.1. Infill Density and Pattern

One of the crucial parameters in MEXM 3D printing is the infill pattern and its density, which also influences the quality and final properties of printed components. Choosing an appropriate infill pattern can be tailored to the specific application of 3D printing [143]. If the goal is to have a lightweight yet durable printout, triangular or honeycomb infills are often used. Different materials may react differently to various infill patterns, affecting the overall print quality and durability. Changing the infill pattern can also affect the printing time. Rosnitschek et al. [144] focused on analyzing the influence of infill density on the geometric and mechanical properties of components printed with 316L stainless steel. They explored the relationship between shape, infill density, effective mechanical properties, and dimensional precision. The results indicate that it is possible to decrease infill density to 50% and 75% without compromising mechanical properties. Moreover, the authors suggest that complete infill can lead to stress accumulation and defects in the structure of printed samples, which may negatively affect mechanical properties. During mechanical properties analysis, tensile tests results revealed variations in the effective Young’s modulus. Lower infill densities (25% and 50%) resulted in lower Young’s modulus compared to samples with higher infill densities (75% and 100%). Additionally, the fracture structure in bending tested samples was more complex for lower infill densities. During bending tests, there was less variance in results for each infill density compared to tensile tests. The effective modulus of elasticity remained consistent across all configurations, ranging between 130 and 142 GPa, with greater variability observed in samples featuring higher infill densities. This parameter emerged as a critical factor influencing the density, shrinkage, and mechanical properties of the examined components. It allows for influencing mass, hardness, and printing time, which can be adjusted to the project’s needs, ensuring high resistance to stresses, internal forces, and other desired functional properties. However, in addition to infill density, the type of infill is also crucial. In the discussed studies, authors applied a single type of infill, while in another study [145] the effect of triangular and fully danse parts patterns, with two feedstock materials—316L stainless steel and copper—on tensile strength was investigated. Two analytical approaches were utilized: experimental tensile testing and finite element analysis (FEA). For 316L stainless steel specimens featuring a triangular infill pattern, tensile strength decreased by 42% relative to fully dense counterparts. Nevertheless, this reduction was offset by significant advantages, including a 34% decrease in mass, 36% cost savings, and a 25% reduction in manufacturing time. Similarly, copper samples with triangular infill experienced a 22% decline in tensile strength alongside a 12% mass reduction; however, production time remained largely unchanged, exhibiting only a 3% decrease. These results underscore the substantial impact of infill geometry on mechanical performance. Specifically, for both 17-4 PH stainless steel and copper, triangular infill configurations led to marked tensile strength reductions while providing benefits in cost efficiency and reduced fabrication duration. When choosing the optimal infill pattern, consider these factors. However, within the sphere of MEXM printing, a range of infill types are available, each offering distinct characteristics tailored to specific applications. Table 11 below presents all infill types used for printing parts with the MEXM method. Akhoundi et al. [146] examined the influence of infill patterns (Table 11) with infill percentages of 20, 50, and 100% on tensile strength, bending, and Young’s modulus.

Parts produced via the MEXM technique frequently demonstrate inferior tensile and bending strengths compared to conventional manufacturing methods such as injection molding and machining. This discrepancy arises primarily from the inherent properties of thermoplastic materials and the relatively weak interlayer adhesion between deposited rasters. The selection of an appropriate printing pattern and infill density markedly influences the mechanical performance of printed components, as experimentally evaluated in this study. The findings suggest that the concentric pattern yields the most favorable tensile and bending properties across varying infill percentages, attributable to the uniform alignment of deposited rasters along the loading axis. In contrast, the Hilbert curve pattern exhibited a notable improvement in mechanical properties at full (100%) infill, likely due to enhanced bonding between rasters and layers facilitated by the sustained high temperature of the strands over short nozzle travel distances. Scanning electron microscopy (SEM) confirmed robust interfacial bonding and well-developed microstructures in samples printed with concentric and Hilbert curve patterns at 100% infill. Conversely, SEM observations of the honeycomb pattern revealed significant voids, which likely contribute to its reduced strength and lower Young’s modulus, particularly at maximum infill.

### 4.2. Orientation of Printed Samples

**Figure 5:**
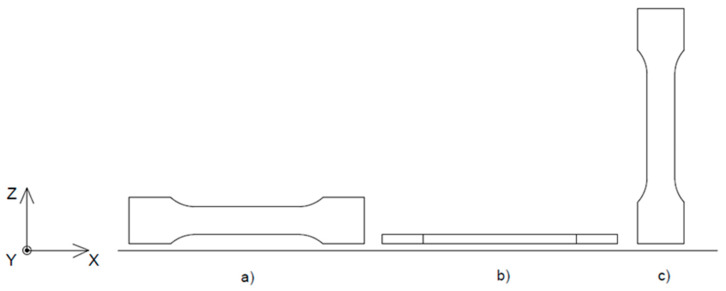
Samples printed in the plane: (**a**) XZ; (**b**) XY; (**c**) ZX.

Another extensively studied factor that affects the quality, durability, and effectiveness of prints, as well as the final properties, is the orientation of the parts in the working space of the devices. In the available studies, the authors compare three different printing orientations, as presented in Figure 5.

Jens et al. [147] delved into the material properties of BASF Ultrafuse 316L stainless steel printed samples, focusing primarily on fracture resistance, porosity, and corrosion resistance. Static tensile testing results revealed two groups of samples (printed in the ZX and XY planes) exhibiting a similar fracture process, with notable differences in tensile strength, elongation at break, and yield strength. Samples printed in the XY plane displayed significantly higher values in these aspects. Charpy’s impact test indicated no significant effect of orientation (print direction) on part properties. The test was conducted at various temperatures, from room temperature to −196 °C, revealing consistent fracture behavior irrespective of print direction. However, samples printed in the XY plane and tested at −196 °C demonstrated a noteworthy increase in impact energy absorption, indicating enhanced impact toughness due to altered crack propagation dynamics. Kurose et al. [148] explored the influence of layering directions on the mechanical properties of 316L stainless steel parts fabricated using MEXM techniques. The study examined the effects of building orientation within the workspace and layer thickness on mechanical properties and dimensional shrinkage. Samples were fabricated using three different layering directions and two distinct layer thicknesses, specifically 0.1 mm and 0.3 mm. Samples printed perpendicular to the layer direction (ZX plane) exhibited the highest tensile strength (453 MPa) and elongation at break (48%), while those printed parallel to the layer direction (XZ and XY planes) displayed inferior mechanical properties. Density examination revealed lower relative roughness in parts with a layer thickness of 0.1 mm. Layering directions minimally affected relative porosity, with the highest recorded value at 7.1%. Linear shrinkage was highest for samples printed parallel to the direction of elongation at break (XZ and XY planes), ranging from 15−17%, compared to 14−15% for samples printed in the ZX plane. Pellegrini et al. [149] demonstrated that samples printed in the ZX orientation exhibited the greatest tensile strength. However, they underscored that it was not the print direction, but rather the density and porosity of the produced samples, that significantly influenced mechanical properties.

**Figure 6:**
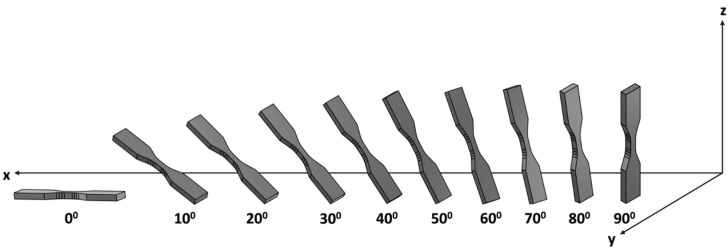
Orientations of printed samples [150].

On the other hand, Alindi et al. [150] broadened their investigations to encompass ten different print orientations (print angles) relative to the printer bed, ranging from 0° to 90° (Figure 6). They utilized 17-4 PH stainless steel for printing.

Samples printed perpendicular to the printing bed (90° orientation) exhibited the lowest tensile strength at maximum load, measuring 440.15 MPa, and an elongation at break of 0.83%. In contrast, specimens printed parallel to the bed (0° orientation) demonstrated an ultimate tensile strength of 947.26 MPa—more than 50% higher than the 90° samples—and an elongation at break of 2.98%, exceeding the latter by over 70%. Based on these findings, the authors recommended printing angles between 0° and 10° to optimize tensile strength. The printed samples were characterized by significant anisotropy, attributed to the presence of surface cracks resulting from insufficient MEXM.

In another study [151], an exploration into the influence of sample orientation on the physical, microstructural, and mechanical attributes of 17-4 PH was conducted across three distinct orientations (depicted in Figure 4), employing an infill ratio of 100%. Analogous to antecedent inquiries, specimens fabricated in the XY plane demonstrated superior surface characteristics, relative sinter density, and tensile strength, exhibiting commendable reproducibility. After sintering, the tensile properties of specimens oriented in the XZ plane were marginally inferior to those in the XY plane but notably superior to those originating from the ZX plane. The tensile characteristics and associated fracture surfaces are attributable to the influences of structural integrity and stress concentration engendered by antecedent void spaces contiguous to the perimeter. Specimens situated in the XZ plane manifested notable deficiencies stemming from the AM process, coupled with vacant interstitial spaces amid perimeter structures, culminating in marked stress concentration and stratification. Discrepancies in tensile properties between specimens produced in the ZX and XY planes predominantly emanate from the inherent dynamics of the AM process—void spaces within the ZX configuration exhibit amplified dimensions and exert a more adverse effect on mechanical properties, as corroborated by fissure patterns evident on the fabricated surface. Additionally, investigations by the authors of studies [152,153,154] validate that specimens printed in the XY orientation surpass counterparts printed in alternative orientations concerning tensile strength.

It is worth noting that similar orientation-dependent behavior is also observed in polymer-based and composite 4D-printed structures, as demonstrated in recent work by Choudhury et al. [155]. The authors showed that print orientation governs not only the mechanical strength but also the programmed shape-morphing and recovery strain in 4D-printed vascular scaffolds. The anisotropy in mechanical properties and functional response was strongly linked to the raster angle and deposition sequence, which is analogous to the behavior observed in metal-polymer MEXM composites. This confirms that orientation-driven anisotropy is a fundamental characteristic of layer-based AM processes, affecting both passive and active performance across different material systems.

## 5. Pre-Processing and Post-Processing

Within the framework of additive manufacturing, pre-processing encompasses a series of procedures conducted to optimize individual printing stages to produce components with desired shapes and properties. These processes entail part design, adaptation to the printer’s build volume, examination and selection of appropriate production parameters, and material preparation, encompassing filament drying before the printing process to eliminate excess moisture absorbed by materials, thereby preventing the formation of gas porosity in the sintered parts.

Most available studies have focused on the characterization of materials printed via MEXM while adhering to the recommended print parameters provided by manufacturers. Johnsson et al. [156] conducted mechanical and microstructural investigations on D2 tool steel printed using MEXM. The findings revealed that the obtained hardness values and tensile test results fell below the manufacturer’s specified reference values. The authors posit that D2 tool steel printed via MEXM holds promise for achieving superior outcomes, yet further experimentation with adjusted process parameters is warranted to mitigate the risk of inadequate MEXM. Lu et al. [157] presented a study utilizing bronze-filled filament for MEXM. The research encompassed microstructural analysis, phase composition, bronze filament characteristics, and assessment of mechanical properties of both printed and sintered samples. Emphasis was placed on the printing process, recommended sintering conditions, and the microstructural features of bronze filament. The investigation disclosed that a sintering temperature below 832 °C yields optimal results, thereby averting significant oxidation reactions. Microstructural examinations unveiled a uniform dispersion of bronze particles within the PLA binder. Mechanical properties, evaluated through three-point bending tests, revealed a bending strength of 27.9 MPa and a Young’s modulus of 1.2 GPa. Fracture observations indicated brittle failure, attributed to the presence of bronze particles serving as stress concentrators. In a study by Hwang et al. [158], novel metal-polymer composite continuous filaments for MEXM were explored. An ABS-based material blended with copper and iron particles was employed in the experiment. Variations in the percentage loading of metal powder and printing parameters, such as temperature and infilling density, were scrutinized to assess their influence on the thermomechanical properties of the filament. The investigation affirmed that the tensile strength of the composites diminishes with increasing metal particle loading, while a higher metal content enhances the thermal conductivity of the composite filament. Other studies [159] have been centered on developing a cost-effective alternative to conventional methods for manufacturing metal parts by employing MEXM based on economically viable AM technology. The study employed material comprising 316L stainless steel powder and a single-component binder, eliminating the necessity for multiple chemical constituents. Samples were fabricated using MEXM technology and subsequently subjected to debinding and sintering processes in a hydrogen atmosphere. The sintered specimens achieved a theoretical density of 93%, predominantly consisted of an austenitic phase, and demonstrated mechanical properties such as a yield strength of 250 MPa, a tensile strength of 520 MPa, and a Vickers microhardness value of 285 HV. The implementation of a single-component binder combined with a reducing hydrogen atmosphere during the final processing phases effectively mitigated oxidation. Furthermore, continuous filaments composed of the single-component binder and stainless steel powder were developed, presenting promising prospects for a cost-effective and sustainable approach to manufacturing metallic components. Thompson et al. [160] conducted investigations on the AM of titanium alloy parts. Crucial stages of production involved controlling thermal processes with precise removal of degradation products and achieving high consolidation during sintering. Optimization of the initial part formation was attained by adjusting extrusion temperatures and infill patterns. Further consolidation of GP was accomplished by heating to 180 °C and compressing under a pressure of 92 MPa, thereby eliminating minor printing defects. Thermal debinding played a pivotal role in removing polymer constituents and preserving the sample’s shape, achieved through gradual heating and an oxygen-free furnace atmosphere. Microstructural analysis revealed a diminished number of grain boundaries in the α phase and Fe compared to samples sintered in an argon atmosphere. This disparity influenced tensile properties, resulting in heightened strength while concurrently reducing ductility due to residual porosity. Despite an elevated oxygen content, the chemical purity of printed and sintered samples remained within acceptable limits. Increasing the vacuum level may facilitate the formation of a more uniform microstructure and improve tensile properties. Sintered titanium alloy specimens displayed slight variations in electrochemical behavior, which were attributed to differences in their chemical composition and surface conditions. Ciorneli et al. [161] presented an experimental analysis concerning the application of low-melting metals such as Sn-58Bi, Sn-9Zn, and Sn-3.5Ag in MEXM. The investigation focused on analyzing the mechanical properties of these materials and their suitability for 3D printing processes. The melting temperature of the materials (Sn-58Bi, Sn-9Zn, Sn-3.5Ag) was below 260 °C. Mechanical properties, including yield strength and elongation at fracture measured during tensile testing, were assessed for each material. Sn-58Bi exhibited the highest yield strength but low elongation at break, whereas Sn-9Zn and Sn-3.5Ag displayed lower yield strength but higher elongation at break. Despite certain technical challenges associated with the mechanical testing of metal filaments, each of the investigated materials (Sn-58Bi, Sn-9Zn, Sn-3.5Ag) appears to be a potentially suitable solution for use as the filament in MEXM printing. Sn-9Zn was selected for further investigation due to favorable mechanical properties and lower cost compared to Sn-58Bi. These studies’ findings suggest the feasibility of employing metal filaments in MEXM 3D printing, opening new avenues for metal utilization in prototyping and production through AM. Pure copper, a less common metal for MEXM [162], was explored by Gonzalez-Gutierrez [163] focusing on producing lightweight copper samples that retain mechanical properties without increasing weight.

The study specifically investigated the influence of infill density and the application of coatings on the debinding behavior and bending performance of sintered components. Results indicated that coated specimens were capable of preserving comparable bending strength while achieving a maximum weight reduction of approximately 23%. However, it was also found that coating samples with infill densities below 100% resulted in a slower debinding process. This indicates that MEXM copper can be effectively utilized in the manufacturing of various parts. Post-processing in the context of metal printing using MEXM is a set of tasks after the printing process itself, aiming to refine, finish, and tailor the printed part to the end user’s expectations. Post-processing includes activities that provide finishing for metal parts, such as removing supports, cleaning prints, grinding, and sand blasting, and also processes that can improve the final properties of metal prints. The main challenge in metal printing using MEXM is the occurrence of porosity, which can significantly reduce the mechanical properties of printed parts. To densify material structures and reduce internal pores, post-sintering thermal treatment is applied. Pellegrini et al. [164] achieved an increase in hardness of 18.9% and 34.3% for two types of 17-4 PH steel using the H900 aging heat treatment process. This process also reduced porosity by 18.4% and 34.3%. Forcellese et al. [110] conducted heat treatment of sintered parts to increase the martensitic grain structure. A solution heat treatment was conducted at 1040 °C for one hour, followed by an aging process at 482 °C for one hour. Beyond conventional heat treatment methods, Bjørheim et al. [154] employed Surface Mechanical Attrition Treatment (SMAT) to enhance surface strain hardening of the samples. This approach enabled a reduction in internal stresses by approximately 700 MPa.

To mitigate internal porosity beyond the intrinsic limits of the MEX process, several non-mandatory post-processing techniques can be selectively applied depending on the target application and required material performance:

1. Surface Mechanical Attrition Treatment (SMAT)—Proven to reduce residual stress and densify the surface layer by severe plastic deformation, SMAT can lower near-surface porosity and improve fatigue performance [154].
2. Hot Isostatic Pressing (HIP)—While expensive and time-consuming, HIP has shown significant effectiveness in eliminating internal pores, particularly for high-performance applications (e.g., aerospace, medical). Selective use of HIP, post-sintering, can raise density from ~95% to >99% [165,166,167,168,169].
3. Post-sintering thermal aging (e.g., H900 for 17-4PH)—This can induce phase transformation and close micropores while increasing hardness. Pellegrini et al. [164] report up to 34% reduction in porosity and 18.9% gain in hardness.
4. Controlled part compression during green part heating—As shown in [160], light pressing of green parts prior to sintering helps remove minor voids and improves layer cohesion.

These techniques, while not required for all applications, can be applied selectively to meet stringent structural or mechanical requirements. In most general-use cases, we recommend SMAT or aging as minimal interventions to reduce porosity without significantly increasing turnaround time.

## 6. Summary and Challenges

The progression of AM technology through metal extrusion is garnering attention due to its comparative advantages over presently employed methods for AM with metals. The principal advantages include reduced equipment costs, operational simplicity, mechanical straightforwardness, and effectiveness in low-volume production. Current studies suggest that MEXM, enabled by economically accessible equipment, holds potential as a feasible manufacturing method for both prototyping and final production components.

Compared to other metal AM techniques such as selective laser melting (SLM) and direct metal laser sintering (DMLS), MEXM offers a lower-cost and more accessible alternative, although it generally yields parts with lower resolution and higher porosity. While SLM and DMLS are well-suited for complex geometries and high-precision components, MEXM excels in rapid fabrication of larger, mechanically robust parts where geometric tolerance requirements are less stringent. In contrast to Metal Injection Molding (MIM), MEXM provides greater flexibility for low-volume production without the need for expensive tooling, making it particularly attractive for prototype development and customized components.

In general, instances have been noted where rapid prototypes composed of high-wear-resistant M2 steel have been manufactured, providing an alternative to MIM for part prototyping [170]. Moreover, several industrial-grade components have been successfully manufactured employing MEXM metal systems [9,171]. However, a significant challenge in this methodology resides in the occurrence of porosity, which can markedly influence the mechanical properties of the final products. For MEXM printing, focused research is imperative to mitigate the incidence of pores within the structure. Heat treatment of sintered parts has demonstrated efficacy in pore reduction. Another potential solution lies in the application of Hot Isostatic Pressing (HIP)—a process renowned for pore minimization and structure densification of metal components. HIP is frequently employed to enhance the structure of parts fabricated using SLM or SLS methods [165,166,167,168,169]. However, there remains a notable deficiency of comprehensive studies aimed at optimizing the HIP process for metallic materials employed in MEXM.

Another key factor influencing the degradation of mechanical properties is the precise adjustment of printing parameters and the attainment of a balance among various parameters to achieve the highest possible density while maintaining desirable properties. In the conducted studies, researchers primarily concentrate on altering layer thickness, printing speed, and temperature. However, there exists a research gap concerning the investigation of alterations in flow parameters, which, if appropriately tuned, could mitigate void spaces between adjacent paths of deposited material, thereby enhancing density within the printed component. Consequently, there exist considerable opportunities for the advancement of materials that would facilitate the future fabrication of mechanically robust MEXM components with enhanced geometrical fidelity. To maximize the cost-effectiveness of AM technology within industrial settings, further experimentation is imperative for process optimization. Significant challenges and future research directions encompass evaluating the viability of binder packing methods and processes to develop filaments that are both optimally dense and suitable for printing. Critical investigations include exploring diverse filament fabrication techniques aimed at achieving metallic density and chemical homogeneity, alongside systematic studies of debinding parameters—such as temperature, heating rate, atmospheric conditions, and duration—to reduce porosity and other defects arising during the debinding stage. Future research should focus on several concrete directions, including real-time monitoring of the debinding process to enable early defect detection and reduce internal flaws; development of hybrid sintering–HIP cycles to combine cost-efficiency with improved densification and mechanical properties; exploration of novel binder chemistries and feedstock formulations to enhance filament homogeneity, printability, and debinding performance; investigation of flow dynamics during extrusion to optimize material deposition; scalable production strategies for larger or multi-material components; and comprehensive fatigue strength and durability testing to certify MEXM parts for demanding industrial applications.

In conclusion, although MEXM technology currently faces certain limitations such as lower resolution and porosity-related defects compared to other AM methods, it offers a cost-effective and flexible alternative well-suited for specific applications including rapid prototyping, low- to medium-volume production, and manufacturing of larger parts with moderate geometric complexity. Its relative ease of operation and reduced material and equipment costs make it attractive for small- to medium-sized enterprises and custom manufacturing scenarios. To adapt MEXM for broader industrial use, continuous research into performance properties, especially fatigue strength and long-term durability, is necessary. These aspects will be the focus of the authors’ future investigations within the scope of their work.

## Figures and Tables

**Table 1:** Type of material and powder content.

Material	Stainless Steel 17-4 PH (Sandvik Osprey)	Stainless Steel 316L (Micro-Melt^®^ Carpenter)	Stainless Steel 316L (BASF Ultrafuse)	Titanium Alloy Ti-6Al-4V (Praxair)	Stainless Steel 17-4 PH (BASF Ultrafuse)	Titania TiO_2_ (US Research)
Powder Content [%]	55	55	88	≤ 0.07	–	65
Carbon (C)	0.01	0.030	15–17.5	–	0.08	–
Chromium (Cr)	15.5–17.5	3–5	–	≤25	≤0.005	≤0.005
Copper (Cu)	3–5	–	0.50	–	–	–
0.15–0.45	–	71.85	62	Bal.	0.25	≤ 1
Manganese (Mn)	0.76	2.00	≤2.0	≤ 1	–	–
Molybdenum (Mo)	0.30	2.25–3.5	Balance	–	–	≤0.005
Nickel (Ni)	3.0–5.0	13–15	–	≤20	Bal.	–
Niobium (Nb)	0.15–0.45	–	0.10	–	–	–
Phosphorus (P)	–	0.040	0.025	≤0.045	–	–
Silicon (Si)	0.84	0.75	≤1.0	–	–	≤0.003
Sulfur (S)	0.030	0.010	–	–	–	≤0.005
Oxygen (O)	–	–	–	≤0.10	0.2	≤0.01
Cobalt (Co)	–	–	–	≤1.0	–	≤0.01
Aluminum (Al)	–	–	–	–	5.50–6.50	≤0.003
Vanadium (V)	–	–	–	3.50–4.50	–	–
Azote (N)	–	–	–	–	0.05	–
Hydrogen (H)	–	–	–	–	0.015	–
Kalium + Natrium (K + Na)	–	–	–	–	–	≤0.005
Magnesium (Mg)	–	–	–	–	≤0.01	–
Wolfram (W)	–	–	–	–	≤0.01	–
Ref.	[65]	[68]	[65]	[69]	[70]	[71]

**Table 2:** Types and composition of binding material.

Material	Producer	Type of Binding	Core Material [50–90%]	Backbone [0–50%]	Additive [0–10%]	Ref.
Stainless steel (17-4PH)	Sandvik Osprey Ltd. (Neath, Wales, UK)	Fiber	Thermoplastic elastomer (TPE)	Grafted Polyolefin	None	[65]
Stainless steel (316L)	Powder: Micro-Melt^®^ 316L, Carpenter Technology Corp. (Reading, PA, USA)	Fiber	Thermoplastic elastomer (TPE)	Grafted Polyolefin	None	[68]
Stainless steel (316L)	BASF Ultrafuse 316LX metal filament (Ludwigshafen, Germany)	Fiber	Polyformaldehyde (POM)	Polypropylene (PP) Dioctyl Phthalate (DOP) Dibutyl Phthalate (DBP) Zinc Oxide	None	[65]
Titanium alloy (Ti-6Al-4V)	Praxair Surface Technologies, (Indianapolis, IN, USA)	Fiber	The raw material is a multi-component polymeric binder. It has a viscosity below 1000 Pa*s and a shear rate below 1000 1/s at a temperature of 240 °C			[72]
Titania (TiO_2_)	US Research Nanomaterials Inc. (Houston, TX, USA)	Powder	Bentonite Powder—Quest White 3411-01			[65]

**Table 3:** Selected printing parameters for metal parts produced by the MEXM method.

Material	Printing Parameters							Ref.
	Nozzle Temperature [°C]	Bed Temperature [°C]	Printing Speed [mm/s]	Nozzle Diameter [mm]	Layer Thickness [mm]	Flow Rate Multiplier [%]	Infill [%] Początek Formularza	
Stainless steel (17-4PH)	215–235	100	15–80	0.4, 0.6	0.2	175	100	[65,88]
Stainless steel (316L)	230–250	90	15–40	0.4	0.10–0.25	125	100	[68,89]
Titanium alloy (Ti-6Al-4V)	190–210	60	50	0.4	0.1	–	100	[72]

**Table 4:** Comparison of dimensions between green and metal parts made of 316L stainless steel.

Printing Orientation	Dimension	Green Part [mm]	Sintered Part [mm]	Shrinkage [%]	Ref.
Parallelly to the printing bed	Y	22.8	19.04	19.73	[94]
	Z	3.75	3.02	25.20	
	X	137.98	115.25	19.72	
Perpendicularly to the printing bed	Y	22.8	19.35	17.81	
	Z	3.78	3.48	8.57	
	X	137.98	114.31	20.70	

**Table 5:** Debinding parameters for available materials.

Material	Solvent Parameters			Thermal Parameters			Ref.
	Atmosphere	Temperature [°C]	Time [h]	Vacuum Furnace [bar]	Temperature [°C]	Time [h]	
Stainless steel (17-4PH)	Cyclohexane	65	24	10^−3^–10^−5^	374–750	1.5	[68]
Stainless steel (316L)	Nitrogen	120	8	–	–	–	[62]
Titanium alloy (Ti-6Al-4V)	Acetone	60	24	–	40	6	[72]

**Table 6:** Sintering parameters of selected alloys [67].

Metal Alloy	Atmosphere	Temperature Process [°C]	The Duration of the Process [h]	Ref.
Stainless steel (17-4PH)	Argon	1200–1350	1.5–4	[72]
Stainless steel (316L)	Vacuum/argonne	1250–1380	1–3	[62,68]
Titanium alloy (Ti-6Al-4V)	Hydrogen	1200–1350	1–3	[65]

**Table 7:** Parameters influencing the properties of parts produced by MEXM.

Stage of the MEXM Process	Parameters Influencing the Final Parts at This Stage	Influence on Final Properties	Ref.
Design	Design dimensions, part geometry	Dimensional accuracy, shrinkage, warping	[108,109]
Material selection	The particle size, morphology, and chemical composition of the powder; drying conditions; granulometric distribution; and the type and formulation of the binder.	Microstructure homogeneity, porosity, sintering behavior	
Raw material preparation	Pressure, temperature, and rotational speed of the extruder, diameter and temperature of the extruder nozzle, viscosity of the granulate, volumetric powder metal content in the raw material, flexural and tensile strength of the filament, shear stresses in the raw material preparation.	Filament strength, extrusion stability, internal defects	
Printing	Bed temperature, cooling speed after deposition, printing speed, type of infill, infill angle, layer adhesion, print orientation, layer width and height, melting temperature of the filament.	Interlayer bonding, dimensional tolerance, surface roughness, porosity	
Post-processing	Cycle temperature and duration, furnace atmosphere and pressure, toxic metal ions, nitrogen or other elements of the sintered part, type of furnace.	Final density, mechanical properties, residual stress, phase composition	

**Table 8:** Mechanical properties of selected MEXM composites.

Mechanical Properties	Material	
	Stainless Steel 316L	Stainless Steel 17-4PH
Tensile strength [MPa]	561	990–1276
Yield strength [MPa]	251	756–1109
Young’s modulus [MPa]	–	191–198
Elongation at break [%]	53	4–6
Charpy Impact Toughness [J/cm^2^]	111	–
Hardness (HV)	128	291–400
Ref.	[89]	[88,110]

**Table 9:** Selected properties of MEXM composite materials compared with other manufacturing techniques.

Mechanical Properties	Material								
	Stainless Steel 316L			Stainless Steel 17-4PH			Titanium Alloy (Ti-6Al-4V)		
	MEX	SLM	Conventional	MEX	SLM	Conventional	MEX	SLM	Conventional
Tensile strength [MPa]	561	633	515	990–1276	1100	1310	–	1020	896
Yield strength [MPa]	251	369	205	756–1109	620	1170	220	943	827
Elongation at break [%]	53	30	60	4–6	16	>10	50	12	15
Hardness	128 HV	140 HV	155 HV	291–400 HV	414 HV	388 HB	–	368 HV	33 HRC
Ref.	[89]	[123,124]	[125]	[88,110]	[126,127]	[128]	[72]	[129,130]	[131]

**Table 10:** Research examining how printing parameters affect the final characteristics of metal parts.

Ref.	Material	Parameters	Results
Godec et al. [133]	Stainless steel 17-4PH	– Nozzle temperature: 210, 220, 235, 250, 260 °C, – Flow rate: 95, 101, 110, 120, 127 mm/s, – Layer height: 0.12, 0.15, 0.20, 0.25, 0.28 mm.	The nozzle size directly affected the geometric parameters of the printed parts. Increasing the nozzle size had a negative influence affecting the dimensional fidelity and surface roughness of the parts, but it contributed to the reduction in pore distribution in the sample’s volume. The increase in nozzle temperature and flow rate reduced the roughness of the samples and had a significant influence on tensile properties.
Fongsamootr et al. [134]	Stainless steel 17-4PH	– Raster angle: 0°/60°, 15°/45°/75°, 30°/90°.	Among the tested raster angles, the 0°/60° configuration demonstrated the highest ultimate strength.
Zhang et al. [135]	The bronze-PLA filament	– Layer height: 0.1, 0.2, 0.3 mm – Nozzle temperature: 220, 230, 240 °C, – Printing speed: 10, 15, 20 mm/s.	The highest part quality and the best geometric mapping were achieved with a layer height of 0.1 mm, a nozzle temperature of 240 °C, and a printing speed of 10 mm/s.
Schumacher and Mortizer [118]	Stainless steel 316L	– Bed temperature: 100, 120 °C – Layer height: 0.15, 0.20 mm, – Nozzle temperature: 230, 240 °C.	No significant influence of the varied parameters on properties such as tensile strength or density was observed.
Caminero et al. [136]	Stainless steel 316L	– Layer height: 0.20, 0.25 mm, – Nozzle diameter: 0.4, 0.6 mm, – Flow rate: 30, 40, 50 mm/s.	The use of a smaller nozzle size contributed to minimizing shrinkage and interlayer porosity. The best density was achieved for the following printing parameters: layer height of 0.20 mm, feed rate of 30 mm/s, and nozzle diameter of 0.6 mm. This combination allowed for the densest sample, although the produced samples exhibited poorer dimensional accuracy.
Moritzier et al. [137]	Stainless steel 316L	For a nozzle diameter of 0.25: – Print speed: 20, 25, 34 mm/s, – Layer height: 0.125 mm, – Extrusion width: 0.22, 0.30, 0.38 mm, – Nozzle temperature: 210–260 °C. For a nozzle diameter of 0.4: – Print speed: 20, 25, 34 mm/s, – Layer height: 0.200 mm, – Extrusion width: 0.35, 0.48, 0.61 mm, – Nozzle temperature: 210–260 °C.	Reduced layer height ensures improved adhesion of the initial layer. The maximum density, reaching 99%, was achieved with the following parameters: nozzle diameter of 0.4 mm and extrusion width of 0.3 mm.
Quatro et al. [138,139]	Stainless steel 316L	– Layer height: 0.1, 0.4 mm, – Nozzle temperature: 170, 240 °C, – Print speed: 20, 50 mm/s.	Increasing the layer height from 0.1 mm to 0.4 mm resulted in a reduction in shrinkage. In contrast, lowering the nozzle temperature from 240 °C to 179 °C did not significantly affect either shrinkage or porosity. Additionally, elevating the print speed from 20 mm/s to 50 mm/s caused an increase in both porosity and shrinkage. Optimal sample density was achieved at a print speed of 20 mm/s combined with a layer height of 0.1 mm.
Hassan et al. [140]	Stainless steel 316L	– Flow rate: 7.5, 12.5, 17.5 mm/s, – Layer height: 0.30, 0.40, 0.50 mm.	An increase in flow rate from 7.5 mm/s to 12.5 mm/s and 17.5 mm/s resulted in elevated porosity and larger grain sizes in the samples. Conversely, variations in layer height from 0.30 mm to 0.40 mm and 0.50 mm did not significantly influence the porosity or grain size.
Vetter et al. [141]	Steel AISI 420	– Printing speed: 20/25, 60/80 mm/s, – Flow rate: 150, 160%, – Nozzle temperature: 120, 145 °C.	The authors found that as the printing speed increased, the samples became denser, possibly because of heightened filament slippage during printing. Conversely, raising the flow rate resulted in even denser samples, whereas a slight change in density was observed with an increase in printing temperature.
Obadimu et al. [92]	Stainless steel 316L	– Layer height: 0.15, 0.20, 0.25 mm, – Printing speed: 30, 35, 40 mm/s, – Raster angle: +45°/−45°/0/90°.	Tensile strength remained largely unaffected by variations in printing speed, whereas layer height and raster angle exhibited notable statistical influence. The optimum tensile strength of 450.3 MPa was achieved at a layer height of 0.20 mm combined with a +45°/−45° raster configuration.
Parenti et al. [142]	Stainless steel 316L	– Nozzle temperature: 90–140 °C, – Raster angle: +45°/−45°.	The best results for the raw milling process were achieved with a nozzle temperature of 120–125 °C.

**Table 11:** Types of infills used in MEXM printing.

Infill Pattern Name	Infill Pattern	Infill Pattern Name	Infill Pattern
Parallel Lines	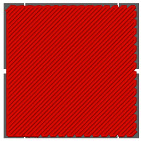	Hilbert Curve	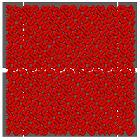
Line	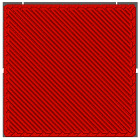	Archimedean Spiral	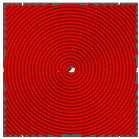
Grid	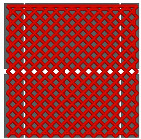	Triangles	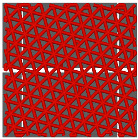
Stars	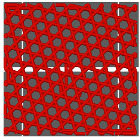	Gyroidal	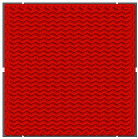
Cubic	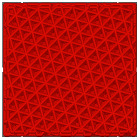	Octagonal Spiral	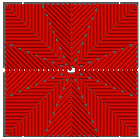
Concentric	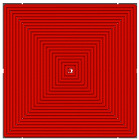	Cubic Infill	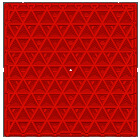
Honeycomb	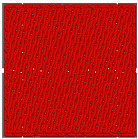	Adaptive Cubic Infill	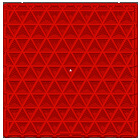
Honeycomb 3D	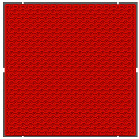		

## Data Availability

Not applicable.
